# Heart failure causally affects the brain cortical structure: a Mendelian randomization study

**DOI:** 10.3389/fnins.2024.1416431

**Published:** 2024-08-01

**Authors:** Tianjiao Meng, Qinwen Fei, Jingying Zhu, Jiayi Gu, Weiyu Li, Xianhong Wu, Gonghua Pan, Tian Lv, Shiqin Chen

**Affiliations:** ^1^Department of Neurology, Zhuji Affiliated Hospital of Wenzhou Medical University, Zhuji, China; ^2^Department of Geriatrics, Zhuji Affiliated Hospital of Wenzhou Medical University, Zhuji, China; ^3^Department of Emergency, Taizhou Hospital, Taizhou, China; ^4^Wenzhou People's Hospital, Wenzhou, China; ^5^The First Clinical Medical Institute of Wenzhou Medical University, Wenzhou Medical University, Wenzhou, China; ^6^Department of Neurology, Second People's Hospital of Yuhuan, Yuhuan, China

**Keywords:** heart failure, Mendelian randomization, cortical structure, heart-brain axis, left ventricular ejection fraction

## Abstract

**Background:**

The effects of heart failure (HF) on cortical brain structure remain unclear. Therefore, the present study aimed to investigate the causal effects of heart failure on cortical structures in the brain using Mendelian randomization (MR) analysis.

**Methods:**

We conducted a two-sample MR analysis utilizing genetically-predicted HF trait, left ventricular ejection fraction (LVEF), and N-terminal prohormone brain natriuretic peptide (NT-proBNP) levels to examine their effects on the cortical surface area (SA) and thickness (TH) across 34 cortical brain regions. Genome-wide association study summary data were extracted from studies by Rasooly (1,266,315 participants) for HF trait, Schmidt (36,548 participants) for LVEF, the SCALLOP consortium (21,758 participants) for NT-proBNP, and the ENIGMA Consortium (51,665 participants) for cortical SA and TH. A series of MR analyses were employed to exclude heterogeneity and pleiotropy, ensuring the stability of the results. Given the exploratory nature of the study, *p*-values between 1.22E−04 and 0.05 were considered suggestive of association, and *p*-values below 1.22E−04 were defined as statistically significant.

**Results:**

In this study, we found no significant association between HF and cortical TH or SA (all *p* > 1.22E−04). We found that the HF trait and elevated NT-proBNP levels were not associated with cortical SA, but were suggested to decrease cortical TH in the pars orbitalis, lateral orbitofrontal cortex, temporal pole, lingual gyrus, precuneus, and supramarginal gyrus. Reduced LVEF was primarily suggested to decrease cortical SA in the isthmus cingulate gyrus, frontal pole, postcentral gyrus, cuneus, and rostral middle frontal gyrus, as well as TH in the postcentral gyrus. However, it was suggested to causally increase in the SA of the posterior cingulate gyrus and medial orbitofrontal cortex and the TH of the entorhinal cortex and superior temporal gyrus.

**Conclusion:**

We found 15 brain regions potentially affected by HF, which may lead to impairments in cognition, emotion, perception, memory, language, sensory processing, vision, and executive control in HF patients.

## 1 Introduction

Heart failure (HF) is now widely acknowledged as a systemic clinical syndrome characterized by insufficient cardiac function linked to multiple organ dysfunction and various co-existing conditions (Doehner et al., 2023). HF often affects the brain and other vital organs, leading to structural and functional abnormalities (Havakuk et al., 2017). Research has shown that individuals with HF may experience a range of neurological impairments, including decreased attention, cognitive dysfunction, dementia, stroke, depression, and autonomic nervous system dysfunction, among others (Florea and Cohn, 2014; Villringer and Laufs, 2021; Doehner et al., 2023). Similarly, the concepts of the heart-brain axis and heart-brain syndrome have become widely accepted (Havakuk et al., 2017; Hooghiemstra et al., 2019).

Focusing on the impact of HF on brain structure, Mueller et al. (2020) found that biomarkers of heart failure, such as left ventricular ejection fraction (LVEF) and N-terminal prohormone brain natriuretic peptide (NT-proBNP) levels, were linked to decreased gray matter density across the entirety of the frontomedian cortex, hippocampus, and precuneus. These observations may reflect structural damage to brain regions associated with cognition. In one prior study utilizing magnetic resonance T2 relaxometry, Woo et al. (2009) discovered that HF patients exhibited differences in regions controlling emotional, cognitive, autonomic, and analgesic functions (temporal, parietal, prefrontal, occipital, insular, cingulate, and ventral frontal cortices; anterior thalamus; caudate nuclei; anterior fornix and hippocampus; hypothalamus, raphé magnus, cerebellar cortex, deep nuclei and vermis; corpus callosum, respectively) compared to controls, suggesting abnormalities in emotional, cognitive, autonomic, and pain functions among HF patients. One review summarising a series of studies achieved similar findings (Alosco and Hayes, 2015). However, observational studies are influenced by many confounding factors; for example, patients with HF often have concomitant advanced age, hypertension, obesity, diabetes, and cardiovascular diseases, and the sample sizes of these studies were usually small. Therefore, the current research results have not been fully validated. Mendelian randomization (MR) can overcome these limitations. The MR approach uses genetic variants to evaluate the causal associations between exposure and outcome variables (Sekula et al., 2016). When applied to large datasets, MR analysis could serve as an excellent method for exploring the impact of HF on brain structure.

In the present study, we utilized human genetic data within the MR framework to investigate the impact of HF on the structure of the brain cortex, specifically the cortical surface area (SA) and cortical thickness (TH), measured using magnetic resonance imaging (MRI). Three sets of HF parameters were used to derive the MR estimates: HF trait, LVEF, and NT-proBNP. Overall, this study provides valuable insights into the heart-brain axis.

## 2 Materials and methods

### 2.1 Study design

**Figure 1:**
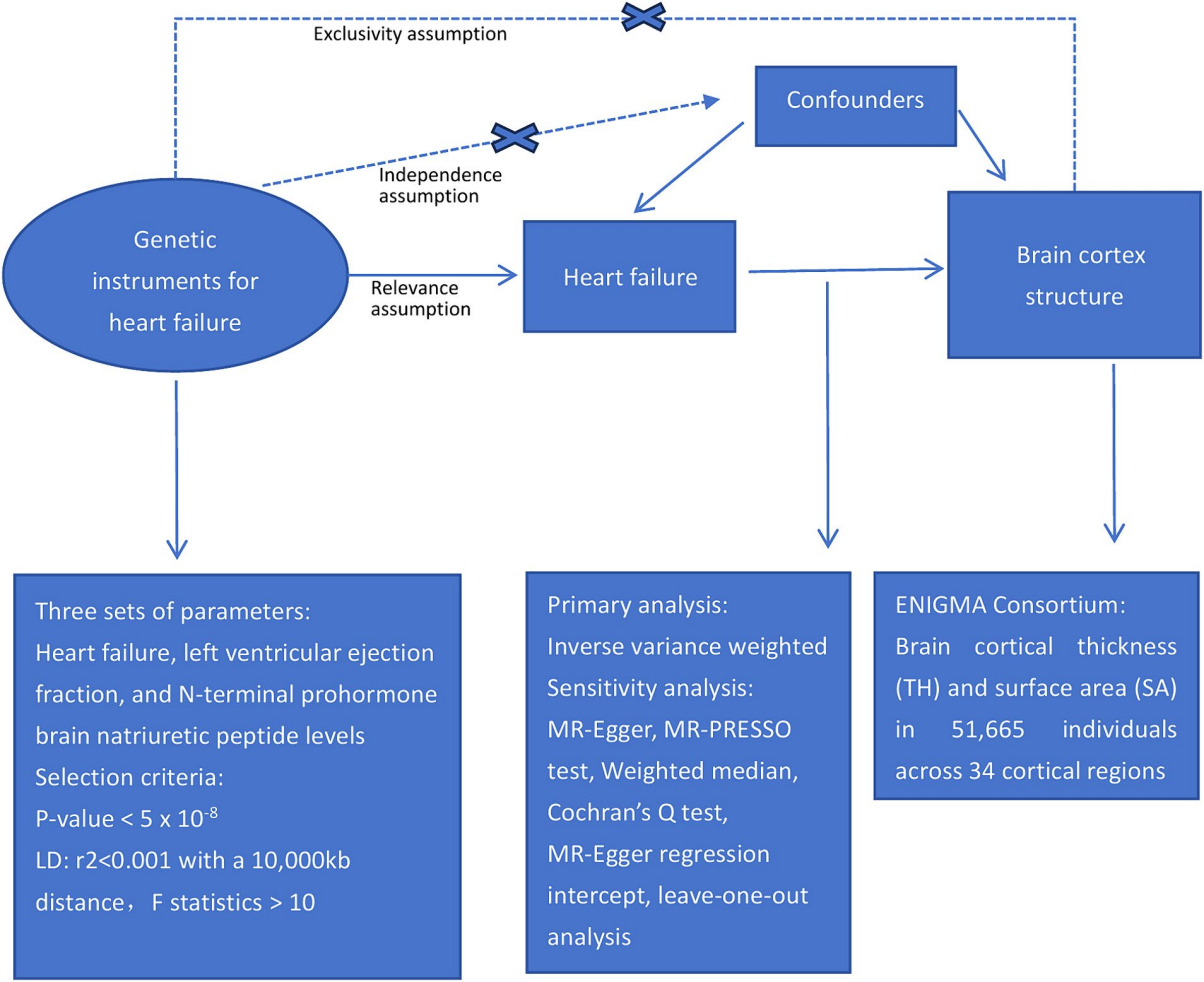
Study design schematic.

This study examined the causal relationships between HF and cortical structures using a two-sample MR approach. Figure 1 presents an overview of the study design.

To ensure a reliable MR method, three fundamental assumptions must be satisfied: first, the genetic instruments should be strongly associated with the exposure; second, the genetic instruments should not be linked to confounding factors; and third, the genetic instruments should only influence the outcomes through the exposures (Richmond and Davey Smith, 2022). The independence of the horizontal pleiotropy, which encompasses the second and third assumptions, can be effectively evaluated using diverse statistical methods (Emdin et al., 2017).

### 2.2 Data sources for HF, LVEF and NT-proBNP

HF data were obtained from a large-scale meta-analysis of genome-wide association studies (GWAS) conducted by Rasooly et al. (2023), encompassing over 90,000 cases and 1 million control individuals of European ancestry. Detailed cohort information is provided in Supplementary Table S1. In their findings, the researchers identified 39 genome-wide significant HF risk variants, including 18 that were previously unreported. Summary statistics related to LVEF were extracted from a study by Schmidt et al. (2023), who conducted a GWAS on 16 cardiac magnetic resonance (CMR) traits measured in up to 36,548 UK Biobank subjects using a thoroughly validated deep-learning approach. Genetic data for NT-pro_BNP were acquired through a GWAS conducted by the SCALLOP consortium (Folkersen et al., 2020), comprising 13 cohorts, totaling 21,758 individuals of European descent; detailed cohort information is provided in Supplementary Table S2.

In summary, utilizing the largest and up-to-date accessible GWAS datasets focused on HF, LVEF, and NT-proBNP, we investigated the potential causal relationships between these HF-related traits and cortical brain structure.

### 2.3 Data source for brain cortex SA and cortex TH

GWAS data related to brain cortical structures were acquired from the ENIGMA Consortium (Grasby et al., 2020). Brain cortical TH and SA measurements were conducted using MRI on 51,665 individuals, predominantly (∼94%) of European descent, across 60 cohorts worldwide. In our study, we specifically employed meta-results derived exclusively from participants of European ancestry, and detailed information about the cohorts is provided in Supplementary Table S3. The 34 cortical regions were delineated based on the Desikan-Killiany atlas, which establishes coarse partitions for the cortex. Regional boundaries were determined in accordance with the gyral anatomy, and were specifically identified between the depths of the sulci (Desikan et al., 2006). Subsequently, these regions were averaged across both hemispheres.

### 2.4 Selection of genetic instruments

To investigate the causal relationship between HF and the cortical structure of the brain, we employed three distinct sets of genetic instruments that signify different facets of heart pathophysiology. Genetic instruments were selected based on the following criteria:

i) A GWAS-correlated *p*-value <5E−08.
ii) Linkage disequilibrium [LD] r2 < 0.001, within a 10,000 kb distance to enhance the independence of single-nucleotide polymorphisms (SNPs).
iii) To ensure result precision and minimize the impact of weak instruments, F statistics (beta^2^/se^2^) were calculated for all SNPs to assess their statistical strength.
iv) Exposure SNPs showing a significant association (*p* < 5E−08) with the outcome were subsequently eliminated.
v) Harmonization was performed to align the alleles of the exposure and outcome SNPs. Palindromic SNPs with incompatible alleles, such as A/G vs. A/C, were also removed.

Overall, 36 index SNPs representing HF (Supplementary Table S4), and 14 index SNPs representing LVEF (Supplementary Table S5) were identified. One index SNP representing NT-pro_BNP was found at a threshold of *p* < 5E−08. However, relying on a single SNP may not fully capture the complexity of the trait (Boehm and Zhou, 2022). Using a looser threshold of p < 5E−06 (Ference et al., 2015; Li et al., 2022), 12 index SNPs for NT-pro_BNP were identified (Supplementary Table S6). With a lowered significance threshold, F statistics were used to assess the potential for weak instrument bias, and all SNPs demonstrated F statistics >10, indicating no bias.

Notably, the lack of overlap among the 36 HF-SNPs in Supplementary Table S4, 14 LVEF-SNPs in Supplementary Table S5, and 12 BNP-SNPs in Supplementary Table S6 indicated the specificity of these instruments.

### 2.5 MR analysis

We employed the inverse variance weighted (IVW) method in the primary analysis to investigate the relationships between heart failure and brain cortical structure. The IVW method is commonly used to estimate causal associations. Results were considered indicative of a suggestive causal relationship between heart failure and brain cortical structures at *p* < 0.05. This method assumes the validity of all genetic variants, making it a valuable approach for MR estimation, despite its susceptibility to pleiotropic bias (Bowden et al., 2015).

### 2.6 Sensitivity analysis

When more than three SNPs were available, sensitivity analyses were conducted using various MR approaches. These included the weighted median, MR-Egger regression, and Mendelian randomization-pleiotropy residual sum and outlier (MR-PRESSO) methods, each of which utilizes distinct assumptions that influence the statistical power. The weighted median approach provided consistent estimates, ensuring reliability when more than half of the weights were derived from valid SNPs (Verbanck et al., 2018). In contrast, MR-Egger analysis is capable of adjusting for pleiotropy and making causal inferences even when all genetic variants exhibit pleiotropic effects (Burgess and Thompson, 2017). Cochran’s Q test was used to assess heterogeneity (Greco et al., 2015). The MR-PRESSO approach was utilized to identify and exclude SNPs with horizontal pleiotropic outliers, minimizing the impact of pleiotropy on causal estimates (Verbanck et al., 2018). Outliers significant at *p* < 0.05 were removed, and the remaining SNPs were subjected to IVW analysis. The presence of pleiotropy in individual SNPs was determined through the MR-Egger regression intercept, with *p*-values >0.05 indicating no horizontal pleiotropy (Bowden et al., 2015). Additionally, leave-one-out analysis was conducted to assess the influence of each SNP on pleiotropy.

### 2.7 Statistical analyses

All analyses were conducted using the Two-Sample MR (Hemani et al., 2018) package in the R environment (ver. 4.3.1; R Development Core Team, Vienna, Austria). Given the exploratory nature of the study. A two-sided *p*-value that passed the Bonferroni-corrected threshold of 1.22E−04 (0.05/408) was defined as statistically significant. Given the exploratory nature of the study, a p-value <0.05 but above 1.22E−04 was considered suggestive of an association.

## 3 Results

**Figure 2:**
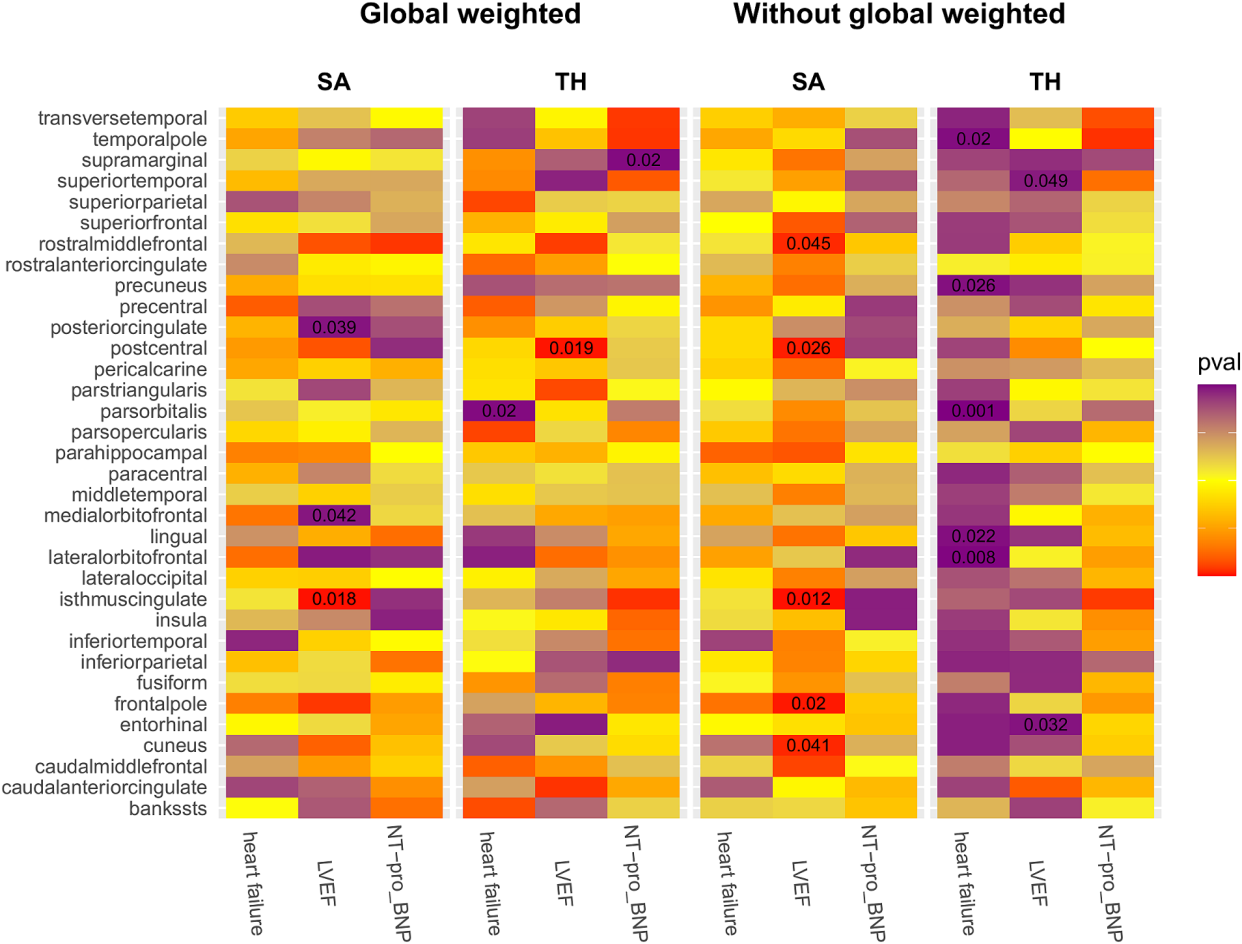
IVW estimates of the impact of heart failure, LVEF, and NT-proBNP on brain cortical structure, defined using magnetic resonance imaging-measured cortical surface area and thickness. Each block’s color indicates the IVW-derived *p*-values from each MRI analysis. *p*-values <0.05 are shown as deep red for positive correlations and deep purple for negative correlations. IVW, Inverse-variance weighted; LVEF, left ventricular ejection fraction; NT-proBNP, N-terminal prohormone brain natriuretic peptide levels; SA, surface area; TH, thickness; Global weighted, global measure as a covariate.

**Figure 3:**
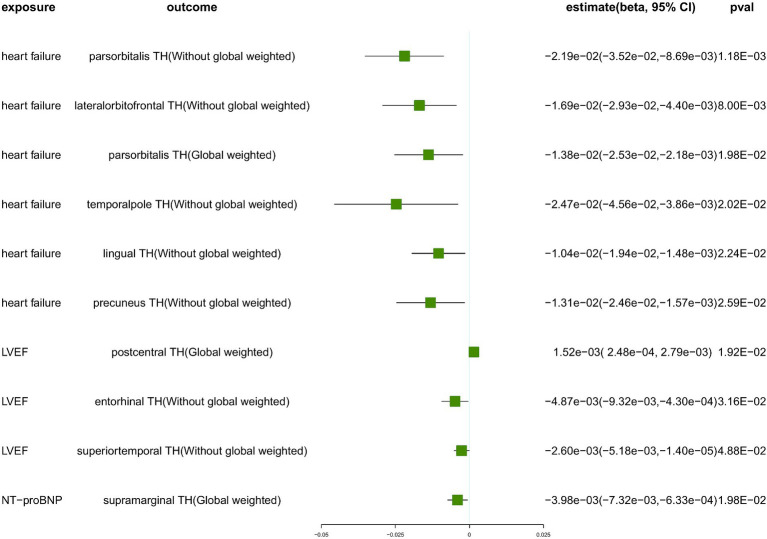
The suggestive results obtained from the inverse-variance weighted evaluation of the impact of heart failure parameters on cortical thickness. SA, surface area; TH, thickness; Global weighted, global measure as a covariate; LVEF, left ventricular ejection fraction; NT-proBNP, N-terminal prohormone brain natriuretic peptide levels.

**Figure 4:**
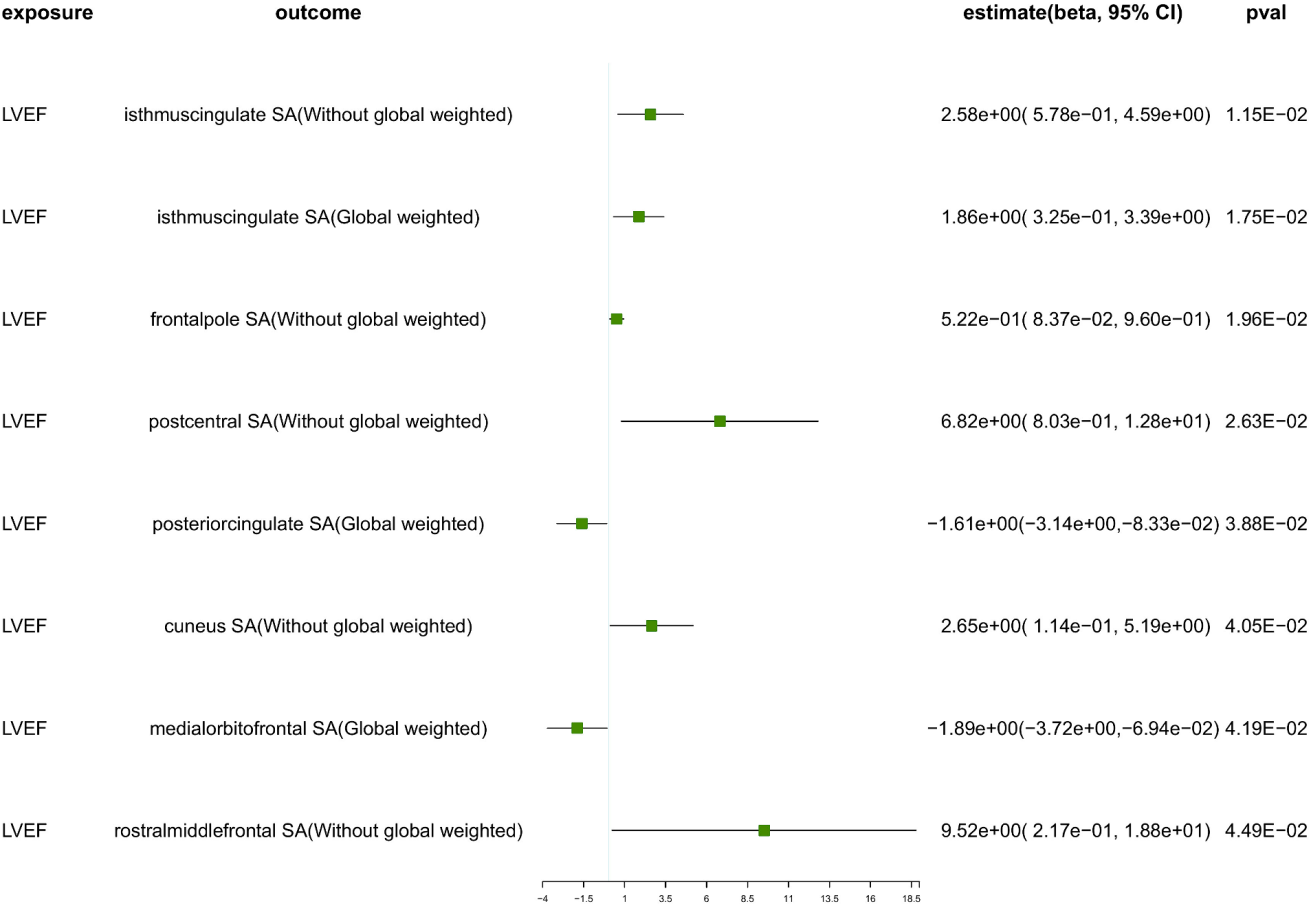
The suggestive results obtained from the inverse-variance weighted evaluation of the impact of heart failure parameters on cortical surface area. SA, surface area; Global weighted, global measure as a covariate; LVEF, left ventricular ejection fraction.

We conducted a comprehensive MR study utilizing genetically predicted HF trait, LVEF, and NT-pro_BNP to examine their effects on 34 functional gyrus SA and cortical TH, both with and without global weight (global measure as a covariate) (Figure 2). The detailed MR results of the primary analysis, comprising 408 outcomes, are listed in Supplementary Table S7. Additionally, we conducted a subgroup analysis based on SA/TH and identified 18 suggestive associations with various gyri (Figures 3, 4).

Genetically predicted HF trait showed no causal relationship with SA, but was suggested to decrease TH (Figure 3) in the following regions: parsorbitalis without global weighting (*β* = − 2.19e−02 mm, 95% CI: − 3.52e−02 mm to −8.69e−03 mm, *p* = 1.18E−03), lateralorbitofrontal without global weighting (*β* = − 1.69e−02 mm, 95% CI: − 2.93e−02 mm to −4.40e−03 mm, *p* = 8.00E−03), global weighted parsorbitalis (*β* = − 1.38e−02 mm, 95% CI: − 2.53e−02 mm to −2.18e−03 mm, *p* = 1.98E−02), temporalpole without global weighting (*β* = − 2.47e−02 mm, 95% CI: − 4.56e−02 mm to −3.86e−03 mm, *p* = 2.02E−02), lingual without global weighting (*β* = − 1.04e−02 mm, 95% CI: − 1.94e−02 mm to −1.48e−03 mm, *p* = 2.24E−02), and precuneus without global weighting (*β* = − 1.31e−02 mm, 95% CI: − 2.46e−02 mm to −1.57e−03 mm, *p* = 2.59E−02). Genetically predicted NT-pro_BNP was suggested to decrease the global weighted supramarginal TH (*β* = −3.98e−03 mm, 95% CI: −7.32e−03 mm to −6.33e−04 mm, *p* = 1.98E−02).

Genetically predicted LVEF was suggested to causally increase the SA of six brain regions and the TH of one brain region (Figures 3, 4), as follows: isthmuscingulate SA without global weighting (*β* = 2.58 mm^2^, 95% CI: 5.78e−01 mm^2^ to 4.59 mm^2^, *p* = 1.15E−02), global weighted isthmuscingulate SA (*β* = 1.86 mm^2^, 95% CI: 3.25e−01 mm^2^ to 3.39 mm^2^, *p* = 1.75E−02), frontalpole SA without global weighting (*β* = 5.22e−01 mm^2^, 95% CI: 8.37e−02 mm^2^ to 9.60e−01 mm^2^, *p* = 1.96E−02), postcentral SA without global weighting (*β* = 6.82 mm^2^, 95% CI: 8.03e−01 mm^2^ to 12.8 mm^2^, *p* = 2.63E−02), cuneus SA without global weighting (*β* = 2.65 mm^2^, 95% CI: 1.14e−01 mm^2^ to 5.19 mm^2^, *p* = 4.05E−02), rostralmiddlefrontal SA without global weighting (*β* = 9.52 mm^2^, 95% CI: 2.17e−01 mm^2^ to 18.8 mm^2^, *p* = 4.49E−02), and global weighted postcentral TH (*β* = 1.52e−03 mm, 95% CI: 2.48e−04 mm to 2.79e−03 mm, *p* = 1.92E−02). Conversely, it was suggested to negatively correlate with the SA of two brain regions and the TH of two brain regions: global weighted posteriorcingulate SA (*β* = −1.61 mm^2^, 95% CI: −3.14 mm^2^ to −8.33e−02 mm^2^, *p* = 3.88E−02), global weighted medialorbitofrontal SA (*β* = −1.89 mm^2^, 95% CI: −3.72 mm^2^ to −6.94e−02 mm^2^, *p* = 4.19E−02), entorhinal TH without global weighting (*β* = −4.87e−03 mm, 95% CI: −9.32e−03 mm to −4.30e−04 mm, *p* = 3.16E−02), and superiortemporal TH without global weighting (*β* = −2.60e−03 mm, 95% CI: −5.18e−03 mm to −1.40e−05 mm, *p* = 4.88E−02).

To ensure the robustness of our findings, we conducted sensitivity analyses using the weighted median and MR-Egger regression methods (Table 1). Notably, the IVW, weighted median, and MR-Egger tests consistently produced results in the same direction. Based on this consistency, we considered the findings to be robust and reliable, and subsequently generated a scatterplot (Supplementary Figures S1−S18). Cochran’s Q-derived *p*-values were all >0.05 (indicating no significant heterogeneity), except for the estimates of heart failure on TH of the lateralorbitofrontal without global weighting and precuneus without global weighting. An outlier (rs4755720) was detected using MR-PRESSO during the estimation of the impact of heart failure on TH of the lateralorbitofrontal without global weighting. Following its exclusion, the analysis was reiterated, and the results were found to be robust, with no significant heterogeneity (IVW: *β* = − 1.37e−02 mm, 95% CI: − 2.46e−02 mm to – 2.70e−03 mm, *p* = 1.46E−02, Cochran’s Q derived p-value = 1.58E−01). As we employed random-effects IVW as the primary outcome, a certain degree of heterogeneity was deemed acceptable (Burgess et al., 2019). All *p*-values (>0.05) derived from the MR-Egger intercept analysis indicated the absence of horizontal pleiotropy. An in-depth scrutiny of our results involved the examination of forest and funnel plots (Supplementary Figures S1−S18). Furthermore, leave-one-out analyses revealed that the estimates were not unduly influenced by any single SNP, further underscoring the robustness of the primary findings.

**Table 1:** Suggestive MR estimates from heart failure, Left ventricular ejection fraction and N-terminal prohormone brain natriuretic peptide levels on genetically predicted cortical structure.

Exposure	Outcome	Method	*p-*val	*β* (95% Confidence intervals)	Q	Cochran’s Q-derived *p* value	Egger_intercept	MR-Egger intercept-derived *p* value
Heart failure	Lateralorbitofrontal TH Without global weighted	Inverse variance weighted	8.00e−03	−1.69e−02 (−2.93e−02, −4.40e−03)	54.77	1.00e−02	4.85e−05	9.61e−01
		MR Egger	4.20e−01	−1.79e−02 (−6.09e−02, 2.51e−02)	54.77	7.34e−03		
		Weighted median	9.27e−02	−1.30e−02 (−2.82e−02, 2.15e−03)				
	Lingual TH Without global weighted	Inverse variance weighted	2.24e−02	−1.04e−02 (−1.94e−02, −1.48e−03)	41.75	1.41e−01	−3.77e−04	5.97e−01
		MR Egger	8.71e−01	−2.53e−03 (−3.29e−02, 2.78e−02)	41.38	1.24e−01		
		Weighted median	4.06e−02	−1.24e−02 (−2.43e−02, −5.32e−04)				
	Parsorbitalis TH Without global weighted	Inverse variance weighted	1.18e−03	−2.19e−02 (−3.52e−02, −8.69e−03)	38.27	2.43e−01	−8.53e−05	9.36e−01
		MR Egger	3.89e−01	−2.02e−02 (−6.54e−02, 2.51e−02)	38.26	2.06e−01		
		Weighted median	7.48e−02	−1.62e−02 (−3.41e−02, 1.62e−03)				
	Precuneus TH Without global weighted	Inverse variance weighted	2.59e−02	−1.31e−02 (−2.46e−02, −1.57e−03)	56.84	6.10e−03	6.60e−05	9.43e−01
		MR Egger	4.76e−01	−1.45e−02 (−5.37e−02, 2.48e−02)	56.83	4.41e−03		
		Weighted median	6.08e−02	−1.32e−02 (−2.70e−02, 6.00e−04)				
	Temporalpole TH Without global weighted	Inverse variance weighted	2.02e−02	−2.47e−02 (−4.56e−02, −3.86e−03)	34.56	3.93e−01	4.27e−04	7.99e−01
		MR Egger	3.63e−01	−3.37e−02 (−1.05e−01, 3.79e−02)	34.48	3.50e−01		
		Weighted median	2.91e−02	−3.41e−02 (−6.48e−02, −3.47e−03)				
	Parsorbitalis TH Global weighted	Inverse variance weighted	1.98e−02	−1.38e−02 (−2.53e−02, −2.18e−03)	44.49	8.74e−02	−2.80e−05	9.76e−01
		MR Egger	5.21e−01	−1.32e−02 (−5.30e−02, 2.66e−02)	44.49	7.01e−02		
		Weighted median	8.83e−02	−1.32e−02 (−2.83e−02, 1.97e−03)				
Left ventricular ejection fraction	Cuneus SA Without global weighted	Inverse variance weighted	4.05e−02	2.65e+00 (1.14e−01, 5.19e+00)	10.59	4.78e−01	7.57e−01	6.83e−01
		MR Egger	8.90e−01	6.88e−01 (−8.85e+00, 1.02e+01)	10.41	4.05e−01		
		Weighted median	2.96e−01	1.83e+00 (−1.60e+00, 5.26e+00)				
	Entorhinal TH Without global weighted	Inverse variance weighted	3.16e−02	−4.87e−03 (−9.32e−03, −4.30e−04)	5.45	9.07e−01	5.83e−05	9.85e−01
		MR Egger	5.60e−01	−5.03e−03 (−2.14e−02, 1.13e−02)	5.45	8.59e−01		
		Weighted median	1.43e−01	−4.31e−03 (−1.01e−02, 1.46e−03)				
	Frontalpole SA Without global weighted	Inverse variance weighted	1.96e−02	5.22e−01 (8.37e−02, 9.60e−01)	8.82	6.39e−01	−5.90e−01	8.13e−02
		MR Egger	3.14e−02	2.06e+00 (4.44e−01, 3.67e+00)	5.06	8.87e−01		
		Weighted median	1.77e−02	7.08e−01 (1.23e−01, 1.29e+00)				
	Isthmuscingulate SA Without global weighted	Inverse variance weighted	1.15e−02	2.58e+00 (5.78e−01, 4.59e+00)	7.96	7.17e−01	7.46e−01	6.04e−01
		MR Egger	8.68e−01	6.43e−01 (−6.73e+00, 8.02e+00)	7.67	6.61e−01		
		Weighted median	2.32e−02	3.17e+00 (4.32e−01, 5.90e+00)				
	Postcentral SA Without global weighted	Inverse variance weighted	2.63e−02	6.82e+00 (8.03e−01, 1.28e+01)	8.07	7.07e−01	−3.94e+00	3.68e−01
		MR Egger	1.62e−01	1.71e+01 (−5.09e+00, 3.92e+01)	7.19	7.08e−01		
		Weighted median	1.64e−01	5.87e+00 (−2.39e+00, 1.41e+01)				
	Rostralmiddlefrontal SA Without global weighted	Inverse variance weighted	4.49e−02	9.52e+00 (2.17e−01, 1.88e+01)	5.89	8.80e−01	−8.55e+00	2.16e−01
		MR Egger	9.92e−02	3.17e+01 (−2.49e+00, 6.59e+01)	4.15	9.41e−01		
		Weighted median	1.88e−01	7.96e+00 (−3.88e+00, 1.98e+01)				
	Superiortemporal TH Without global weighted	Inverse variance weighted	4.88e−02	−2.60e−03 (−5.18e−03, −1.40e−05)	16.2	1.34e−01	2.19e−03	2.38e−01
		MR Egger	1.10e−01	−8.31e−03 (−1.76e−02, 9.63e−04)	14	1.73e−01		
		Weighted median	2.46e−01	−1.94e−03 (−5.22e−03, 1.34e−03)				
	Isthmuscingulate SA Global weighted	Inverse variance weighted	1.75e−02	1.86e+00 (3.25e−01, 3.39e+00)	10.86	4.55e−01	2.07e+00	8.04e−02
		MR Egger	2.48e−01	−3.52e+00 (−9.16e+00, 2.11e+00)	7.08	7.18e−01		
		Weighted median	9.93e−02	1.88e+00 (−3.57e−01, 4.13e+00)				
	Medialorbitofrontal SA Global weighted	Inverse variance weighted	4.19e−02	−1.89e+00 (−3.72e+00, −6.94e−02)	10.34	5.00e−01	−5.68e−01	6.66e−01
		MR Egger	9.06e−01	−4.17e−01 (−7.17e+00, 6.34e+00)	10.14	4.29e−01		
		Weighted median	1.40e−01	−1.90e+00 (−4.42e+00, 6.24e−01)				
	Posteriorcingulate SA Global weighted	Inverse variance weighted	3.88e−02	−1.61e+00 (−3.14e+00, −8.33e−02)	5.78	8.88e−01	−6.71e−01	5.42e−01
		MR Egger	9.64e−01	1.32e−01 (−5.50e+00, 5.76e+00)	5.38	8.64e−01		
		Weighted median	9.39e−02	−1.70e+00 (−3.69e+00, 2.89e−01)				
	Postcentral TH Global weighted	Inverse variance weighted	1.92e−02	1.52e−03 (2.48e−04, 2.79e−03)	11.99	3.65e−01	4.21e−04	6.60e−01
		MR Egger	8.75e−01	4.10e−04 (−4.57e−03, 5.39e−03)	11.75	3.02e−01		
		Weighted median	7.24e−02	1.52e−03 (−1.38e−04, 3.17e−03)				
N-terminal prohormone brain natriuretic peptide levels	Supramarginal TH global weighted	Inverse variance weighted	1.98e−02	−3.98e−03 (−7.32e−03, −6.33e−04)	8.38	3.97e−01	2.25e−04	8.06e−01
		MR Egger	2.92e−01	−4.99e−03 (−1.36e−02, 3.59e−03)	8.3	3.07e−01		
		Weighted median	4.51e−02	−4.63e−03 (−9.16e−03, −1.02e−04)				

SA Global weighted, Total Surface Area included as a covariate; TH global weighted, Average Thickness included as a covariate; Without global weighted, without a global measure as a covariate.

## 4 Discussion

To investigate the impact of HF on the cortical structure, we conducted a MR analysis from three perspectives of genetically predicted HF: HF trait, pathophysiology, and blood biomarkers. Eighteen suggestive associations involving 15 brain regions were identified. Our results found that the HF trait and elevated NT-proBNP were not associated with cortical SA, but were suggestively associated with reduced cortical TH in the pars orbitalis, lateral orbitofrontal cortex, temporal pole, lingual gyrus, precuneus, and supramarginal gyrus. Interestingly, reduced LVEF was primarily suggested to decrease cortical SA in the isthmus cingulate gyrus, frontal pole, postcentral gyrus, cuneus, and rostral middle frontal gyrus, and TH in the postcentral gyrus, but was suggested to increase the SA and TH of certain gyri, including the posterior cingulate gyrus, medial orbitofrontal cortex, entorhinal cortex, and superior temporal gyrus. These brain regions govern higher-order cognitive functions, such as cognition, emotion, perception, memory, and executive control, offering a neuropathological structural basis that elucidates the neurological functional impairments commonly observed in HF patients. Again, these results support the existence of the heart-brain axis and further provide novel insights into its function.

In our study, we found potential structural changes in brain regions associated with cognition and emotion (entorhinal cortex, posterior cingulate gyrus, pars orbitalis, lateral orbitofrontal cortex, temporal pole, frontal pole, precuneus, supramarginal gyrus, isthmus cingulate gyrus, medial orbitofrontal cortex, and rostral middle frontal gyrus). These changes are believed to be related to the cognitive and emotional impairments observed in patients with HF. Cognitive dysfunction is highly prevalent among individuals with HF, with an estimated 25–75% of patients experiencing some level of impairment (Hajduk et al., 2013; Ampadu and Morley, 2015; Doehner et al., 2018). Past meta-analyses and observational studies have consistently shown that individuals with HF demonstrate an overall decline in cognitive performance and specific deficits in areas such as executive functioning, psychomotor speed, and verbal memory compared to those without a history of HF (Hammond et al., 2018; Connors et al., 2021). Our study further found a potential decrease in cortical TH and SA in brain regions associated with higher cognitive function, which may impair the patients’ executive function, verbal memory, and other higher cognitive abilities, consistent with previous research findings. Moreover, neuroimaging studies have revealed that patients with HF exhibit adverse structural brain changes that are associated with cognitive impairment. These include reduced hippocampal volumes (Woo et al., 2015), medial temporal lobe atrophy (Vogels et al., 2007), posterior cingulate cortex atrophy (Almeida et al., 2013), and myelin breakdown (Kumar et al., 2011). Additionally, cortical volume reductions have been observed in various lobes, including the frontal (Almeida et al., 2012), temporal (Woo et al., 2003), and parietal (Almeida et al., 2012) lobes, along with an increased white matter lesion burden (Stegmann et al., 2021). The results of these studies are broadly consistent with our findings, but there are also some differences. We did not observe a decrease in cortical TH and SA in the medial temporal lobe. In contrast, we found that a decrease in LVEF was suggestively associated with an increase in cortical TH in the entorhinal cortex, as well as a probable increase in SA in the posterior cingulate gyrus and medial orbitofrontal cortex. In HF patients, the compensatory mechanism of cerebral autoregulation remains intact or even enhanced, and it can compensate for the hypoperfusion caused by HF (Ogoh et al., 2022). This might explain our findings that while a more exquisitely sensitive cortical area undergoes atrophy and volume reduction in the setting of HF, another less vulnerable area might increase in size and volume to temporarily compensate. Further studies are warranted to confirm these findings.

Emotional disorders, particularly depression and anxiety, are commonly observed in patients with HF, with approximately 20–50% experiencing anxiety and 20–45% suffering from depression (Tsabedze et al., 2021; Rashid et al., 2023). Depression is further associated with an increased risk of mortality in this patient population (Sbolli et al., 2020). Studies focusing on brain structural changes in HF patients with emotional disorders have found an overlap in brain regions associated with emotion and cognition (Woo et al., 2009; Pan et al., 2013; Suzuki et al., 2016). Hippocampal damage is also associated with depression. This association has been confirmed to some extent in animal model experiments (Suzuki et al., 2015). We observed no structural changes in the medial temporal lobe, which includes the hippocampus. This absence of a change may be due to the methodological approach of averaging measurements across both hemispheres, considering that earlier studies have indicated that the right hippocampus is primarily affected by atrophy in HF patients (Woo et al., 2015). Nonetheless, we found potential structural abnormalities in the frontal, temporal, and parietal lobes, particularly in the gyri, that are linked to emotional processing, which supports the results of previous research.

Our study suggested that structural changes in the postcentral gyrus, cuneus, lingual gyrus, and superior temporal gyrus—regions involved in language, sensory processing, and vision—are influenced by HF. Limited research has been conducted on structural brain changes related to speech, sensation, and vision in patients with HF. A study on abnormal autonomic responses to the Valsalva maneuver revealed bilateral damage to the posterior central gyrus, supporting our findings (Song et al., 2018). The absence of gray matter in the cuneus and damage to the lingual gyrus have also been observed in other studies, consistent with our own research findings (Almeida et al., 2012; Park et al., 2016). Contrary to previous research (Almeida et al., 2013), our study found a potential correlation between reduced LVEF and increased TH in the superior temporal gyrus. Further investigation is necessary to validate these findings.

From a mechanistic perspective, HF is associated with reduced cardiac output, inflammatory processes, neurohormoral imbalances, nutritional factors, and damage to brain structures through affected bioelectric and endocrine signaling pathways (Havakuk et al., 2017; Maroofi et al., 2022). These mechanisms are systemic, suggesting that brain damage is widespread rather than confined to specific brain regions. This notion is further supported by our study findings that revealed potential alterations in 15 brain regions.

Advantages of Our Study: First, MR significantly improves traditional methods by addressing confounding factors, reverse causality, and the ethical limitations of randomized controlled trials (RCTs) (Davies et al., 2018). MR uses genetic instruments, specifically, SNPs, which are strongly associated with exposure factors and randomly assorted according to Mendel’s second law of inheritance, similar to an RCT (Lawlor et al., 2008). This methodology significantly reduces confounding and robustly assesses causal relationships between exposure and outcome variables (Lawlor et al., 2008). Second, the large sample size in GWAS provides high statistical power, enhancing the robustness and reliability of our findings (Davies et al., 2018). Finally, our study comprehensively examines HF through three aspects: HF trait, LVEF, and NT-proBNP levels. These parameters represent the clinical phenotype, pathophysiological phenotype, and blood biomarker phenotype of HF, respectively. By incorporating these diverse aspects into an MR analysis of cortical structures, we deepen our understanding of the heart-brain axis.

Our findings not only reveal the potential neuropathological structural changes associated with brain damage caused by HF, but also provide further support for the cognitive, emotional, linguistic, and sensory impairments observed in these patients. Furthermore, our MR analysis fulfilled the assumptions of MR analyses, namely, relevance and independence (Davies et al., 2018). However, this study has several limitations which must be acknowledged. First, owing to the scarcity and heterogeneity of GWAS data on cortical gyral structures, we were unable to validate our current findings using GWAS results from other cortical regions. Second, we discovered inconsistencies between the alterations in the four gyral structures and those reported previously, warranting further research for confirmation. However, the underlying mechanism requires further investigation. Third, we found no significant association between HF and cortical TH or SA (all *p* > 1.22E−04). Our findings suggest potential causal relationships, but further studies with larger sample sizes are needed. Finally, it is important to mention that the data was primarily sourced from individuals of European descent. Therefore, additional GWAS studies involving participants from diverse racial backgrounds are necessary to validate the generalizability of our results.

## 5 Conclusion

This is the first comprehensive MR analysis to reveal a potential causal relationship between HF and cortical structures. Overall, we found 15 brain regions likely affected by HF, which may lead to impairments in cognition, emotion, perception, memory, language, sensory processing, vision, and executive control in patients with HF. These findings provide valuable insights into the potential neurological consequences of HF, and further highlight the importance of considering brain health in its management.

## Data availability statement

The datasets presented in this study can be found in online repositories. The names of the repository/repositories and accession number(s) can be found at: All data are publicly available. Summary statistics for LVEF and NT-proBNP were obtained from the GWAS Catalog (https://www.ebi.ac.uk/gwas/downloads/summary-statistics) with the identifiers GCST90268125 and GCST90012082, respectively. SNPs data for heart failure were extracted from Rasooly's study (DOI: 10.1038/s41467-023-39253-3). GWAS summary data for cortical structure were downloaded from the ENIGMA consortium website (http://enigma.ini.usc.edu/research/download-enigma-gwas-results). The MR analysis code can be found at https://github.com/shiqinchen/data.

## Ethics statement

The studies involving humans were approved by the ethical issues related to the GWAS datasets involved in this study have all been approved by the local ethics committee. The studies were conducted in accordance with the local legislation and institutional requirements. The participants provided their written informed consent to participate in this study.

## Author contributions

TM: Writing – original draft. QF: Writing – original draft. JZ: Writing – original draft. JG: Writing – original draft. WL: Writing – original draft, Data curation. XW: Writing – original draft, Data curation. GP: Writing – original draft, Methodology. TL: Writing – review & editing, Project administration, Conceptualization. SC: Writing – review & editing, Methodology, Conceptualization.
