# Supplementary material for: Heart failure causally affects the brain cortical structure: a Mendelian randomization study
Source: Front Neurosci. 2024 Aug 1;18:1416431. doi: 10.3389/fnins.2024.1416431 (PMC11324602; doi:10.3389/fnins.2024.1416431)

**Supplementary Information**

Supplementary Tables

Table S1. Descriptions of study cohorts participating in Rasooly's study (heart failure)

Table S2. Overview of participating cohorts in the SCALLOP CVD1 study

Table S3. Descriptions of study cohorts participating in Grasby's study

Table S4. 36 index SNPs represented genetically predicted heart failure

Table S5. 14 index SNPs represented genetically predicted Left ventricular ejection fraction

Table S6. 12 index SNPs represented genetically predicted N-terminal prohormone brain natriuretic peptide levels

Table S7. The detailed MR results in primary analysis

Supplementary Figures

Figure S1. Scatter, Forest, Funnel, and Leave-one-out plots of genetically predicted heart failure effects on lateral orbitofrontal cortical thickness (TH) without global weighted

An outlier (rs4755720) was excluded


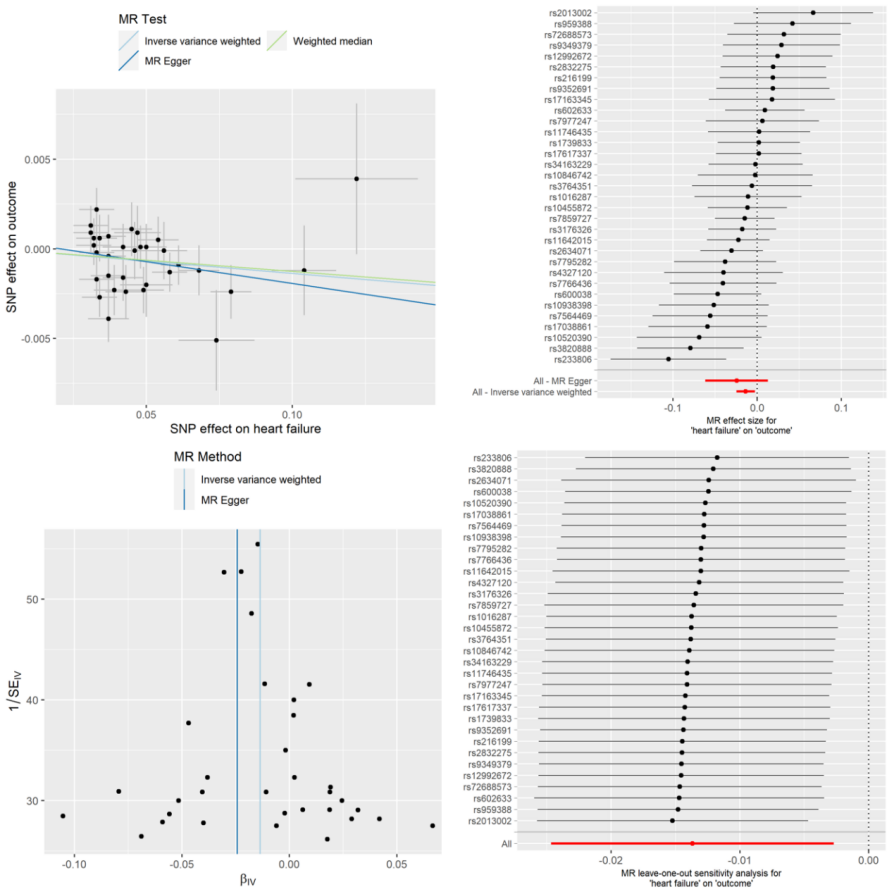


Figure S2. Scatter, Forest, Funnel, and Leave-one-out plots of genetically predicted heart failure effects on lingual cortical thickness (TH) without global weighted


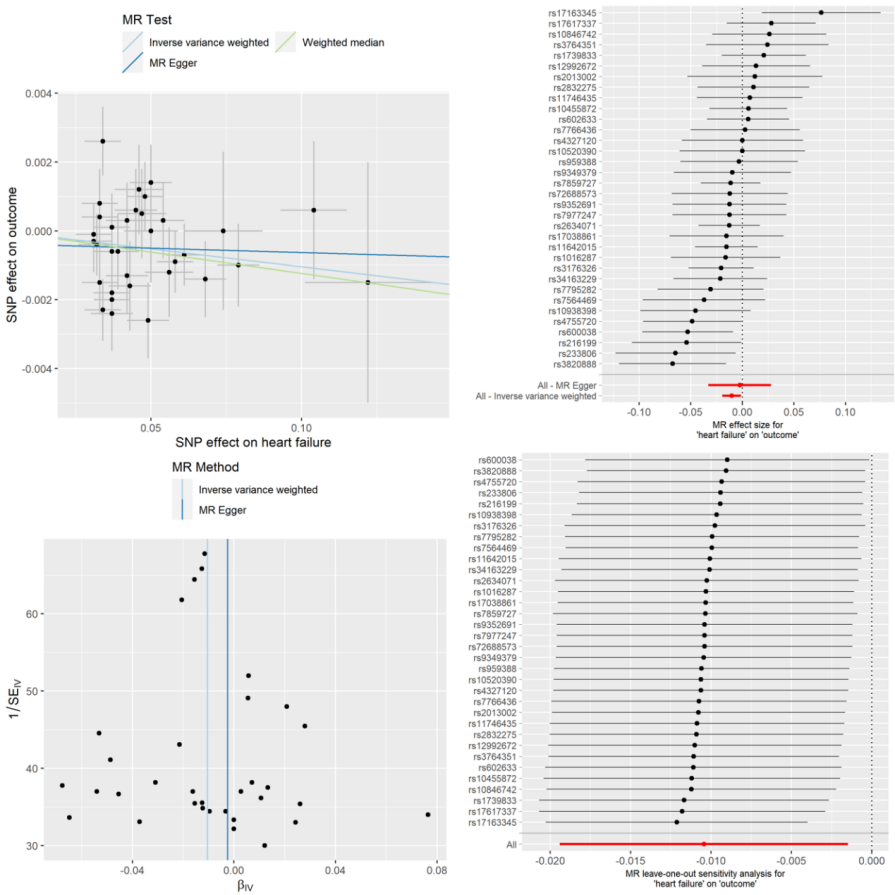


Figure S3. Scatter, Forest, Funnel, and Leave-one-out plots of genetically predicted heart failure effects on parsorbitalis cortical thickness (TH) without global weighted


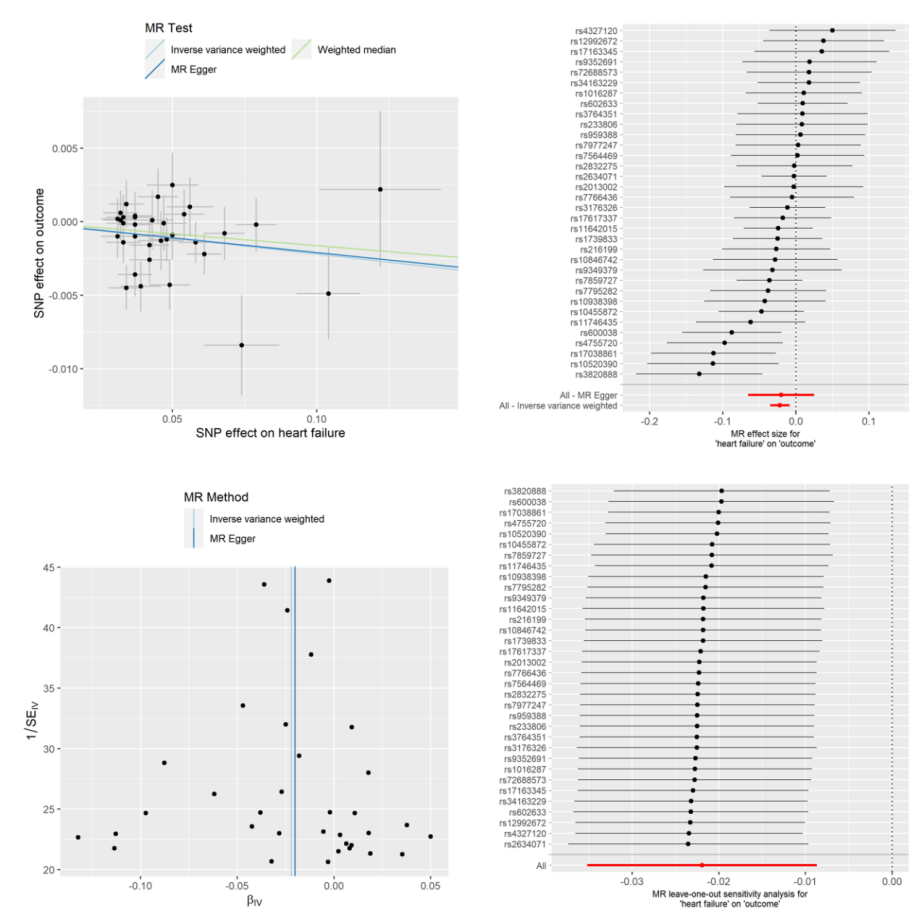


Figure S4. Scatter, Forest, Funnel, and Leave-one-out plots of genetically predicted heart failure effects on precuneus cortical thickness (TH) without global weighted


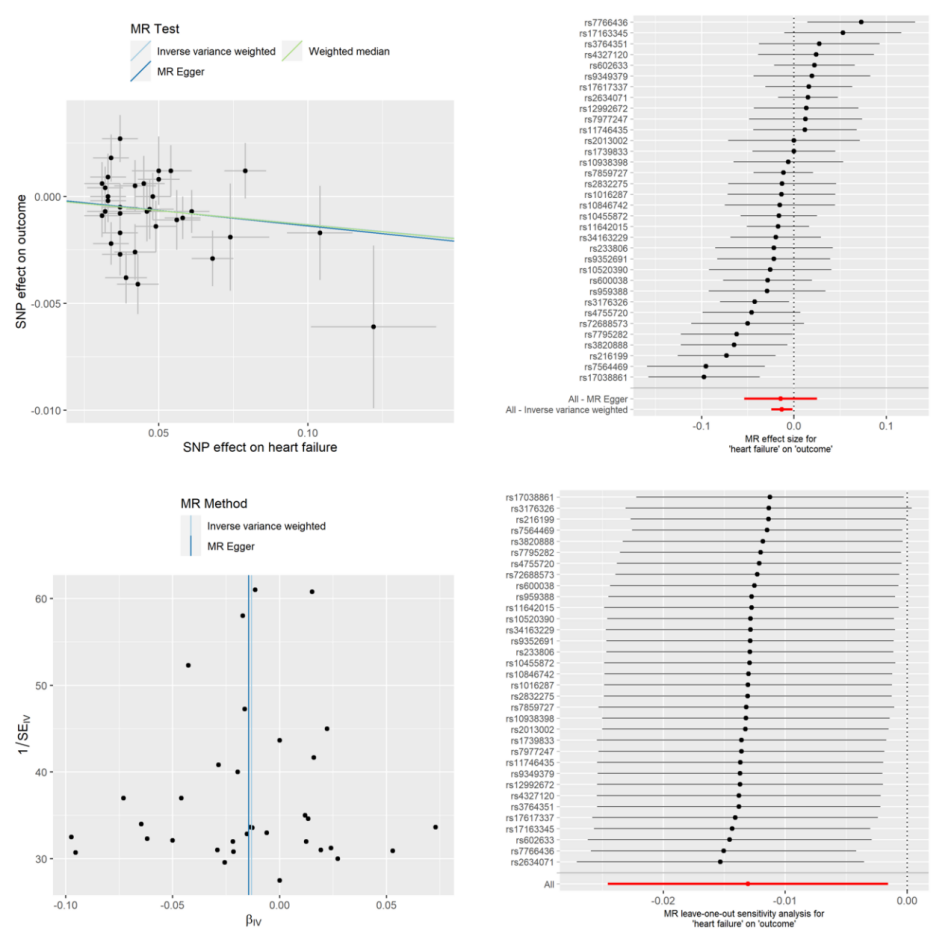


Figure S5. Scatter, Forest, Funnel, and Leave-one-out plots of genetically predicted heart failure effects on temporalpole cortical thickness (TH) without global weighted


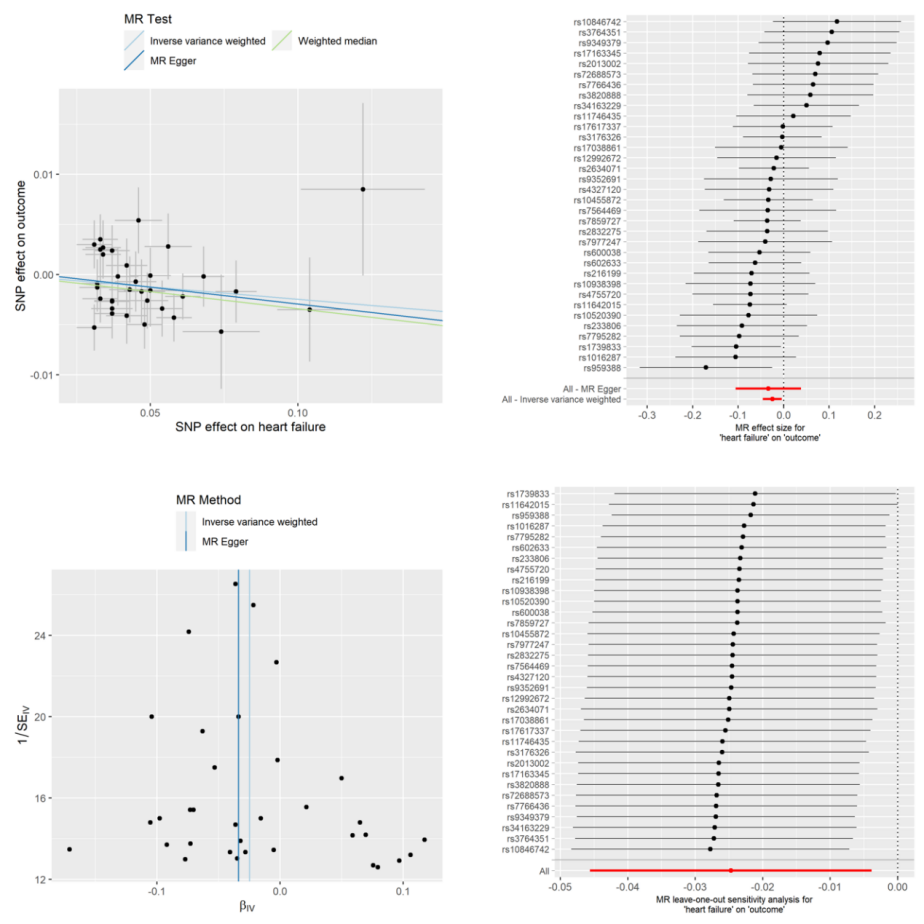


Figure S6. Scatter, Forest, Funnel, and Leave-one-out plots of genetically predicted heart failure effects on parsorbitalis cortical thickness (TH) global weighted


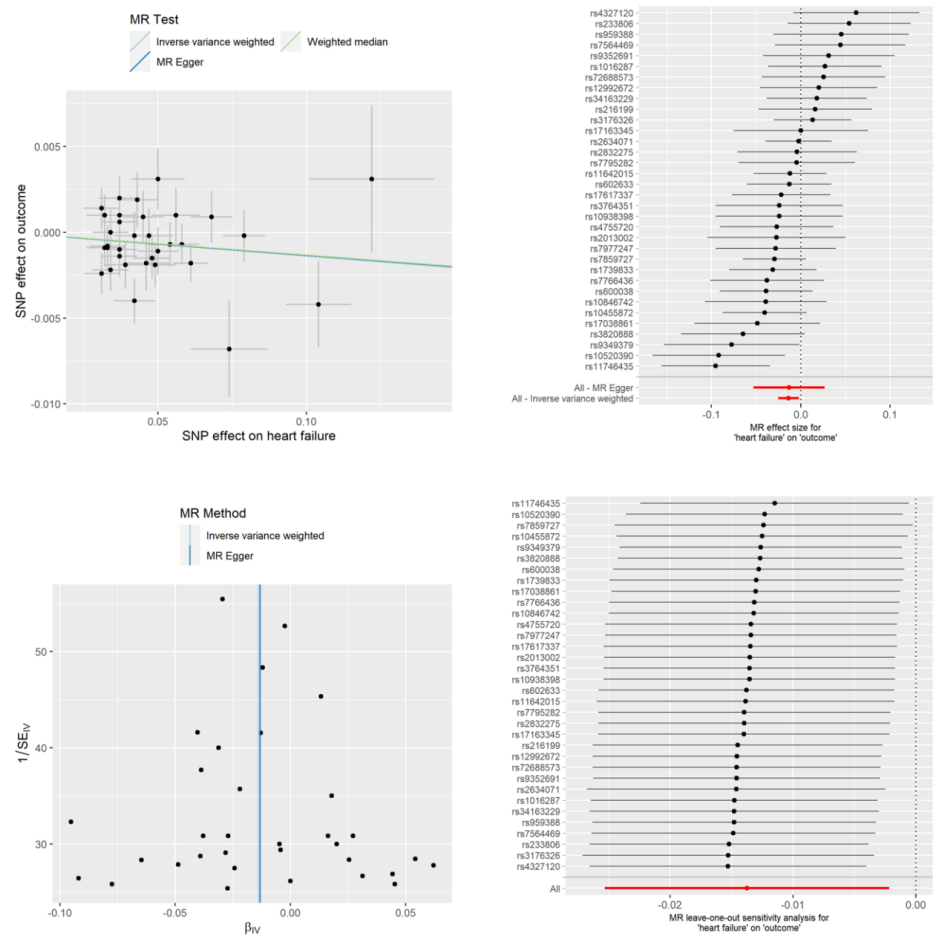


Figure S7. Scatter, Forest, Funnel, and Leave-one-out plots of genetically predicted Left ventricular ejection fraction effects on cuneus surface area (SA) without global weighted


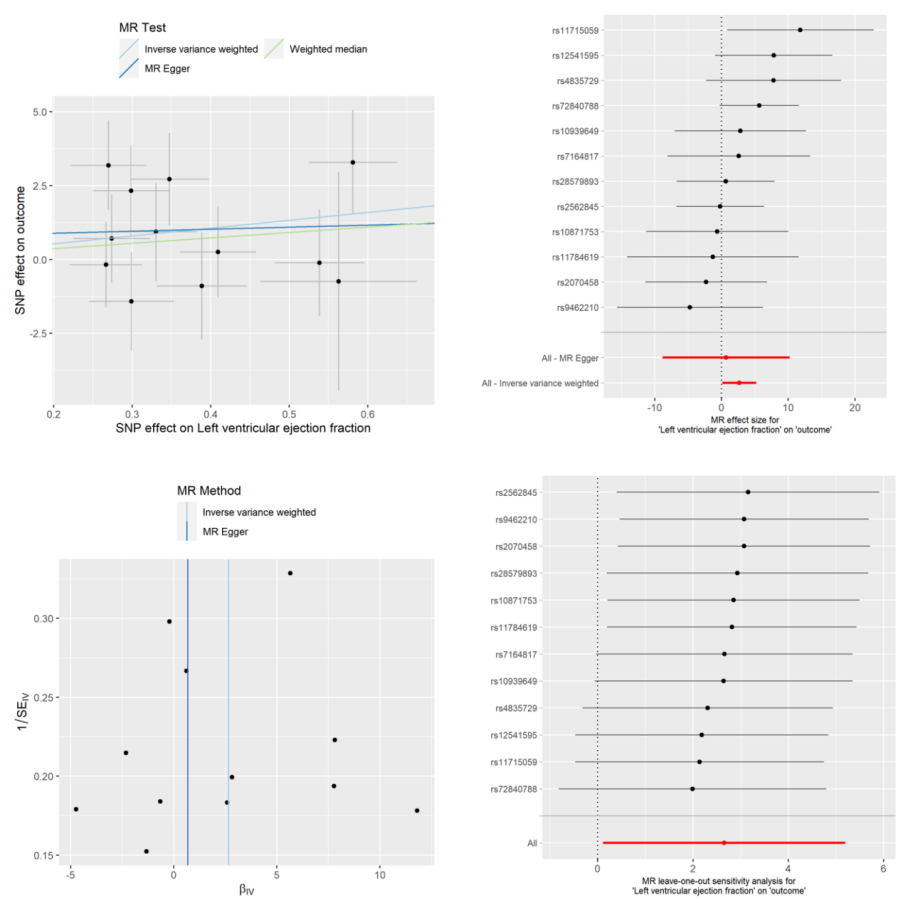


Figure S8. Scatter, Forest, Funnel, and Leave-one-out plots of genetically predicted Left ventricular ejection fraction effects on entorhinal cortical thickness (TH) without global weighted


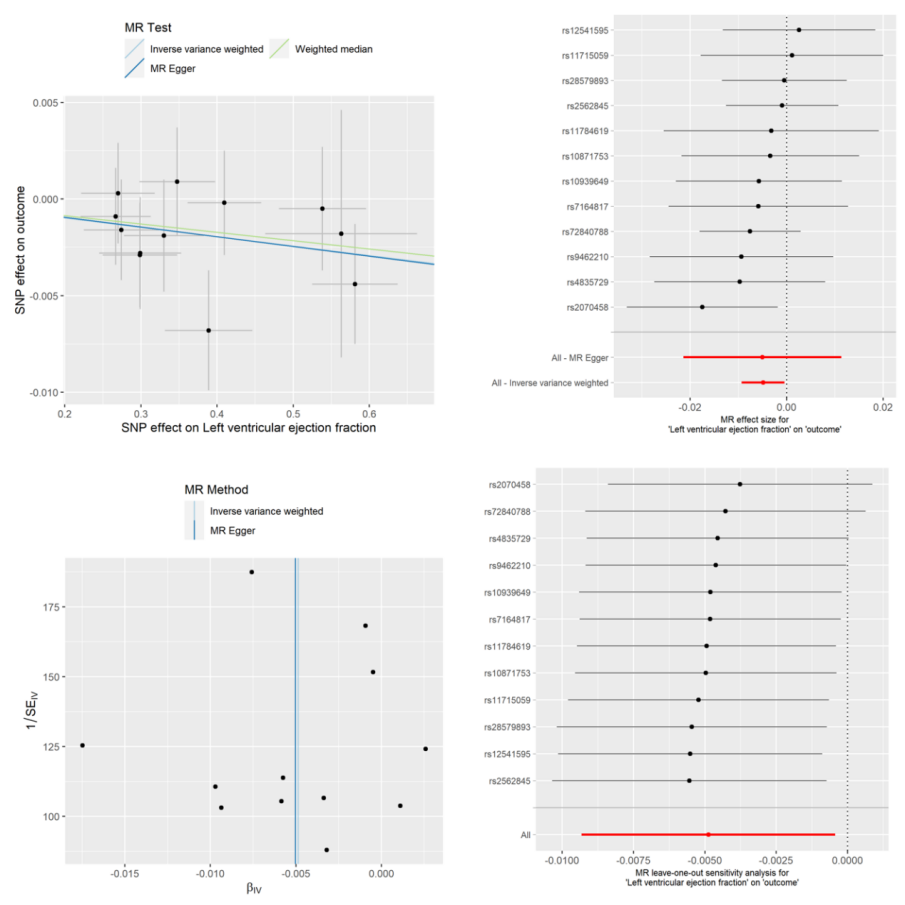


Figure S9. Scatter, Forest, Funnel, and Leave-one-out plots of genetically predicted Left ventricular ejection fraction effects on frontalpole surface area (SA) without global weighted


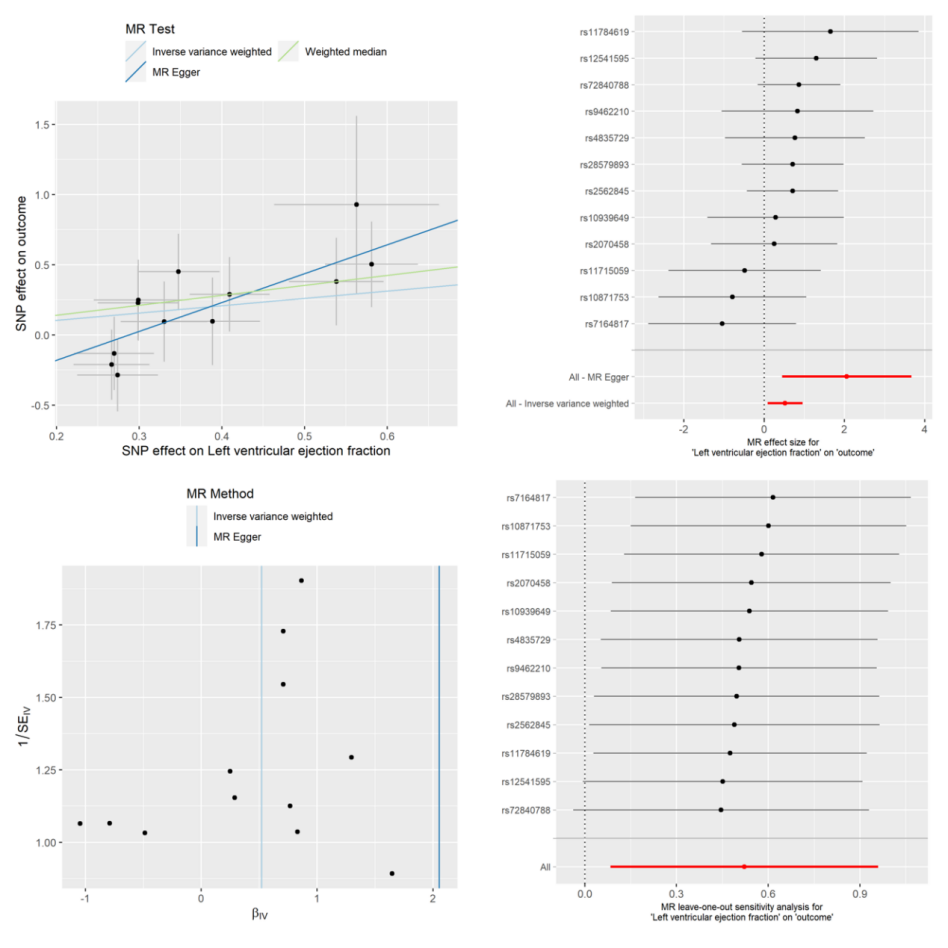


Figure S10. Scatter, Forest, Funnel, and Leave-one-out plots of genetically predicted Left ventricular ejection fraction effects on isthmuscingulate surface area (SA) without global weighted


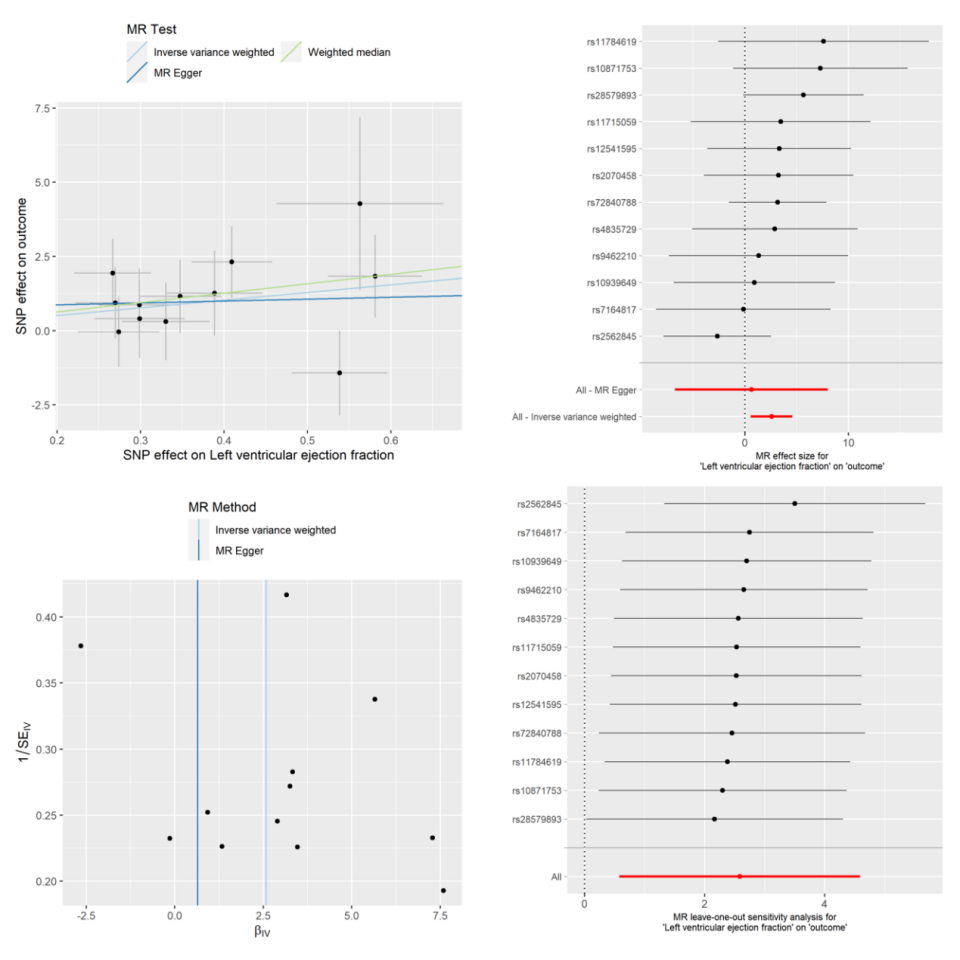


Figure S11. Scatter, Forest, Funnel, and Leave-one-out plots of genetically predicted Left ventricular ejection fraction effects on postcentral surface area (SA) without global weighted


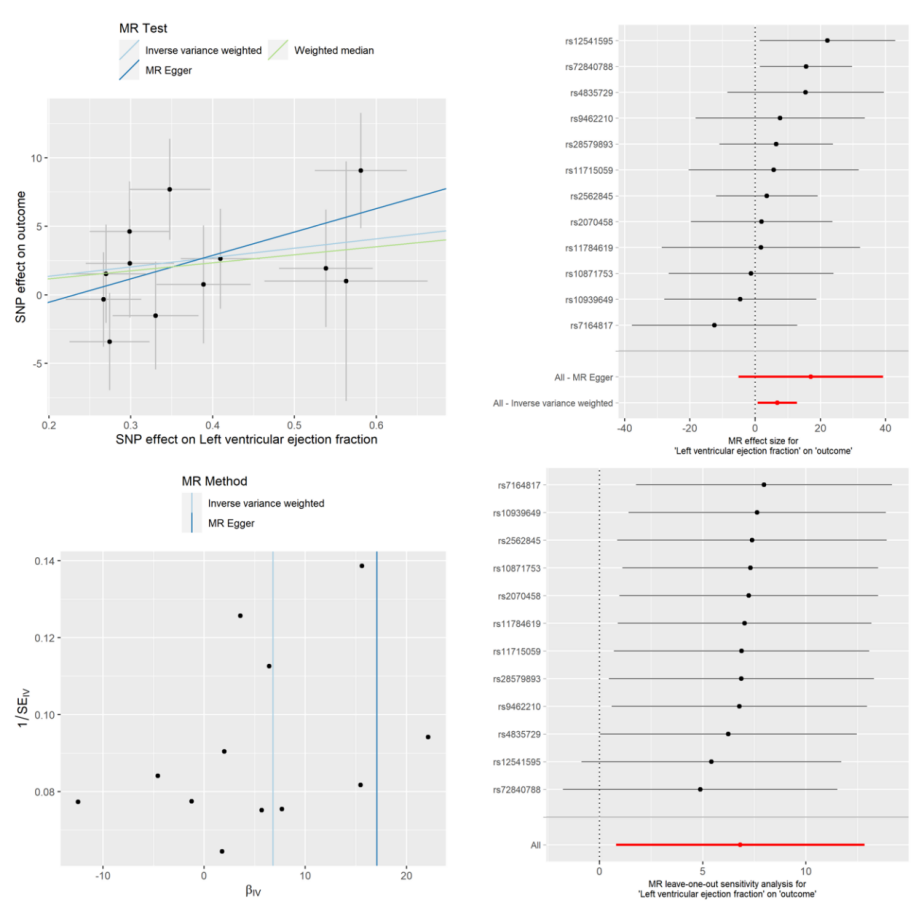


Figure S12. Scatter, Forest, Funnel, and Leave-one-out plots of genetically predicted Left ventricular ejection fraction effects on rostralmiddlefrontal surface area (SA) without global weighted


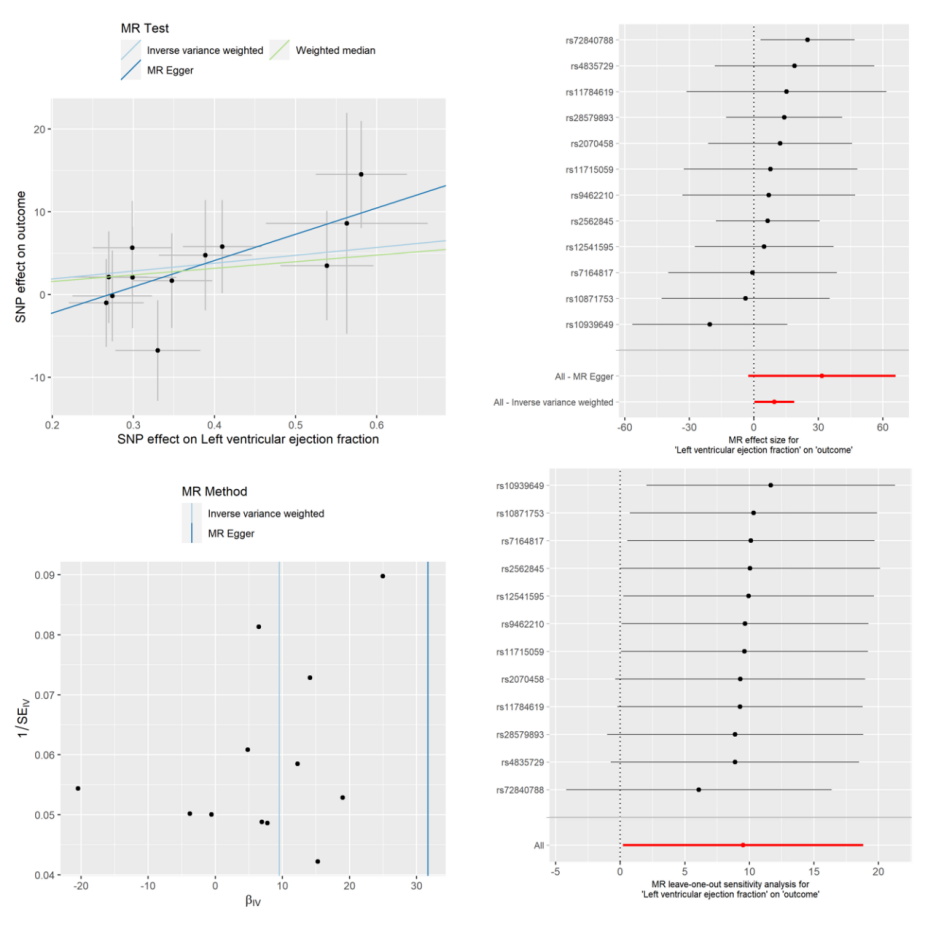


Figure S13. Scatter, Forest, Funnel, and Leave-one-out plots of genetically predicted Left ventricular ejection fraction effects on superiortemporal cortical thickness (TH) without global weighted


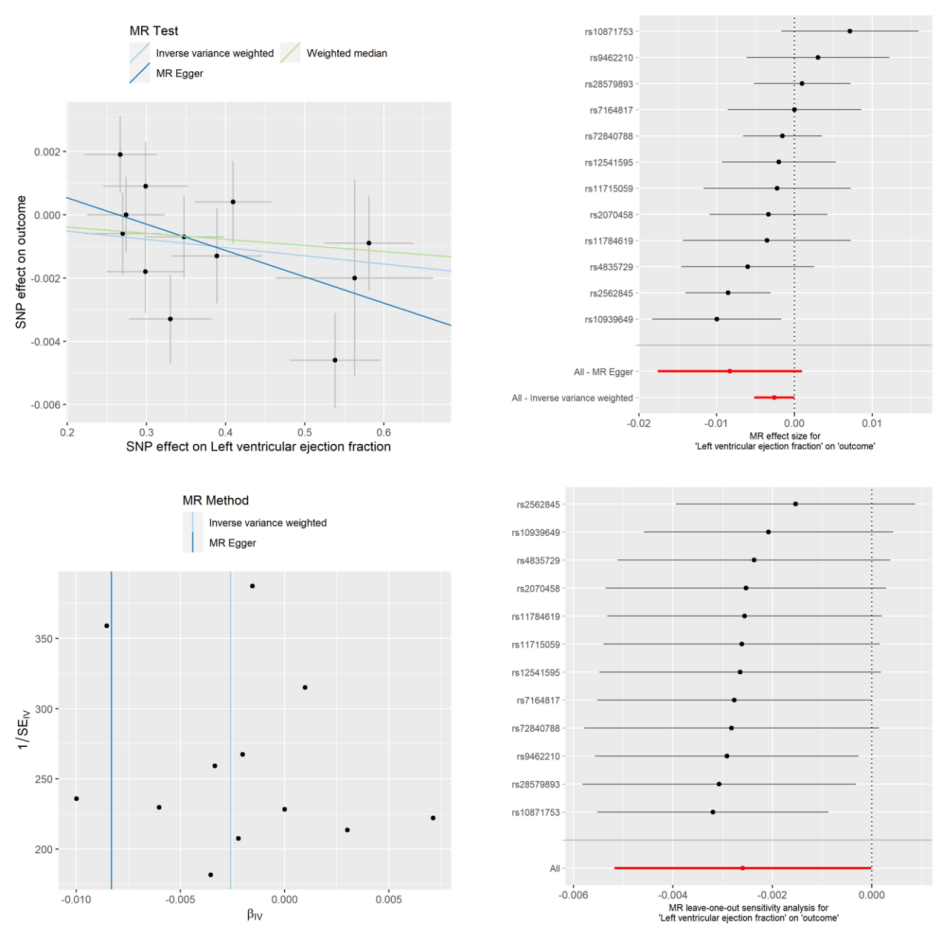


Figure S14. Scatter, Forest, Funnel, and Leave-one-out plots of genetically predicted Left ventricular ejection fraction effects on isthmuscingulate surface area (SA) global weighted


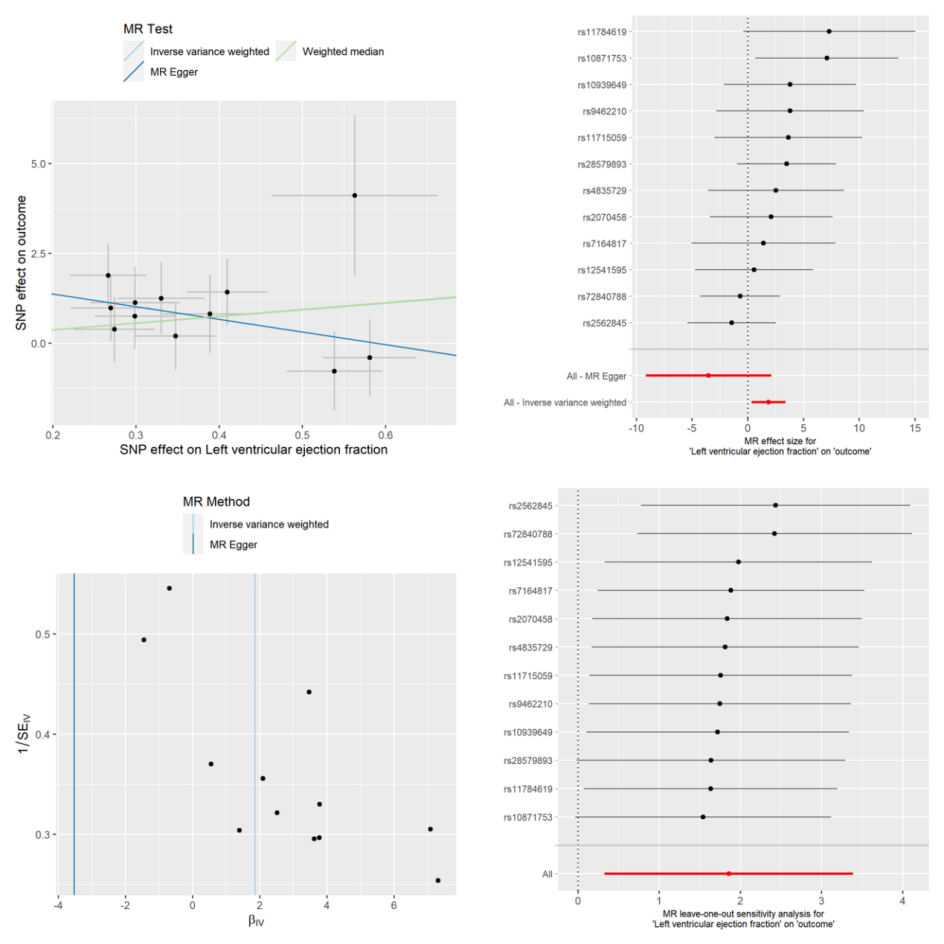


Figure S15. Scatter, Forest, Funnel, and Leave-one-out plots of genetically predicted Left ventricular ejection fraction effects on medialorbitofrontal surface area (SA) global weighted


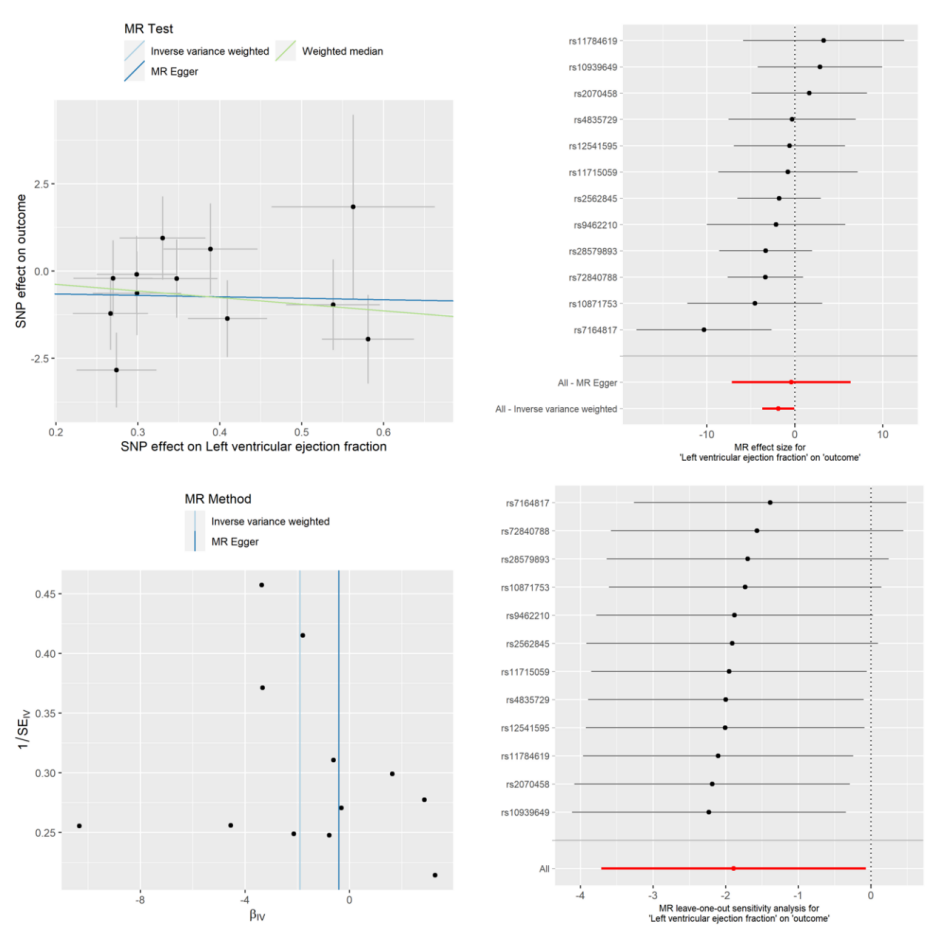


Figure S16. Scatter, Forest, Funnel, and Leave-one-out plots of genetically predicted Left ventricular ejection fraction effects on posteriorcingulate surface area (SA) global weighted


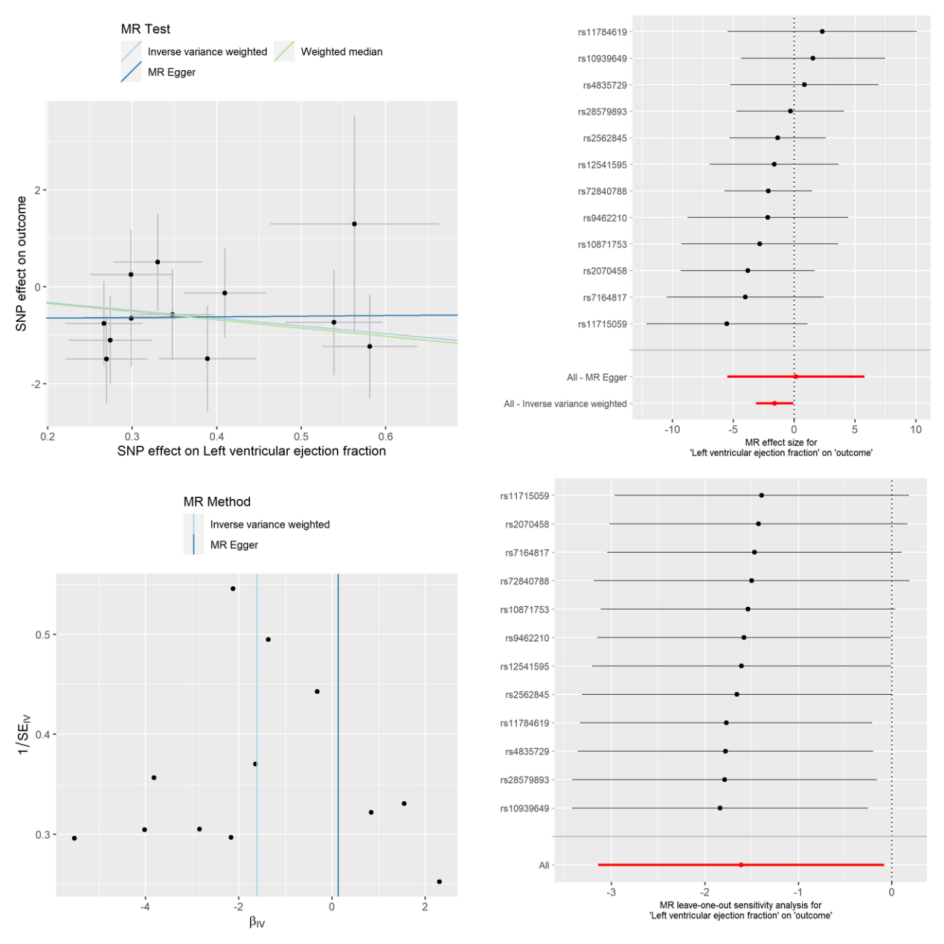


Figure S17. Scatter, Forest, Funnel, and Leave-one-out plots of genetically predicted Left ventricular ejection fraction effects on postcentral cortical thickness (TH) global weighted


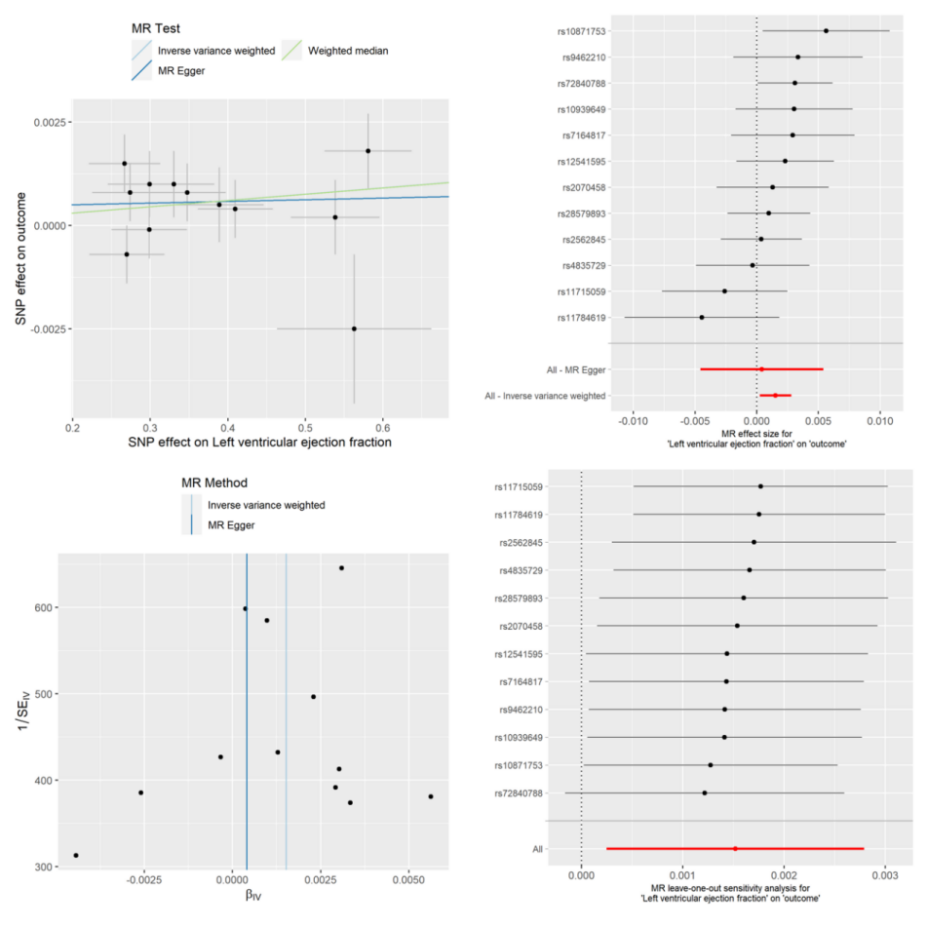


Figure S18. Scatter, Forest, Funnel, and Leave-one-out plots of genetically predicted N−terminal prohormone brain natriuretic peptide levels effects on supramarginal cortical thickness (TH) global weighted


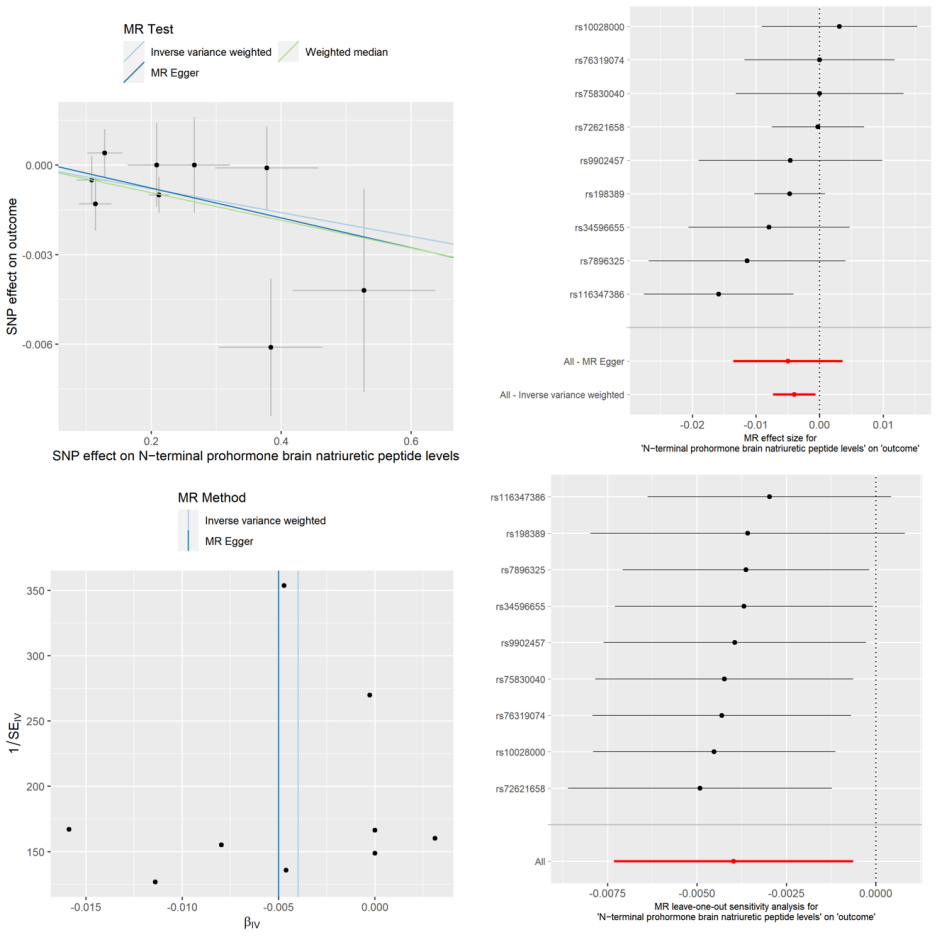

Supplement: Supplementary file 2 [file Data_Sheet_2.docx]
